# Population structure drives cultural diversity in finite populations: A hypothesis for localized community patterns on Rapa Nui (Easter Island, Chile)

**DOI:** 10.1371/journal.pone.0250690

**Published:** 2021-05-12

**Authors:** Carl P. Lipo, Robert J. DiNapoli, Mark E. Madsen, Terry L. Hunt

**Affiliations:** 1 Department of Anthropology, Environmental Studies Program, Harpur College of Arts and Sciences, Binghamton University, Binghamton, NY, United States of America; 2 Department of Anthropology, University of Washington, Seattle, WA, United States of America; 3 The Honors College and School of Anthropology, University of Arizona, Tucson, AZ, United States of America; Institute of Evolutionary Biology, Pompeu Fabra University, SPAIN

## Abstract

Understanding how and why cultural diversity changes in human populations remains a central topic of debate in cultural evolutionary studies. Due to the effects of drift, small and isolated populations face evolutionary challenges in the retention of richness and diversity of cultural information. Such variation, however, can have significant fitness consequences, particularly when environmental conditions change unpredictably, such that knowledge about past environments may be key to long-term persistence. Factors that can shape the outcomes of drift within a population include the semantics of the traits as well as spatially structured social networks. Here, we use cultural transmission simulations to explore how social network structure and interaction affect the rate of trait retention and extinction. Using Rapa Nui (Easter Island, Chile) as an example, we develop a model-based hypothesis for how the structural constraints of communities living in small, isolated populations had dramatic effects and likely led to preventing the loss of cultural information in both community patterning and technology.

## Introduction

**Fig 1:**
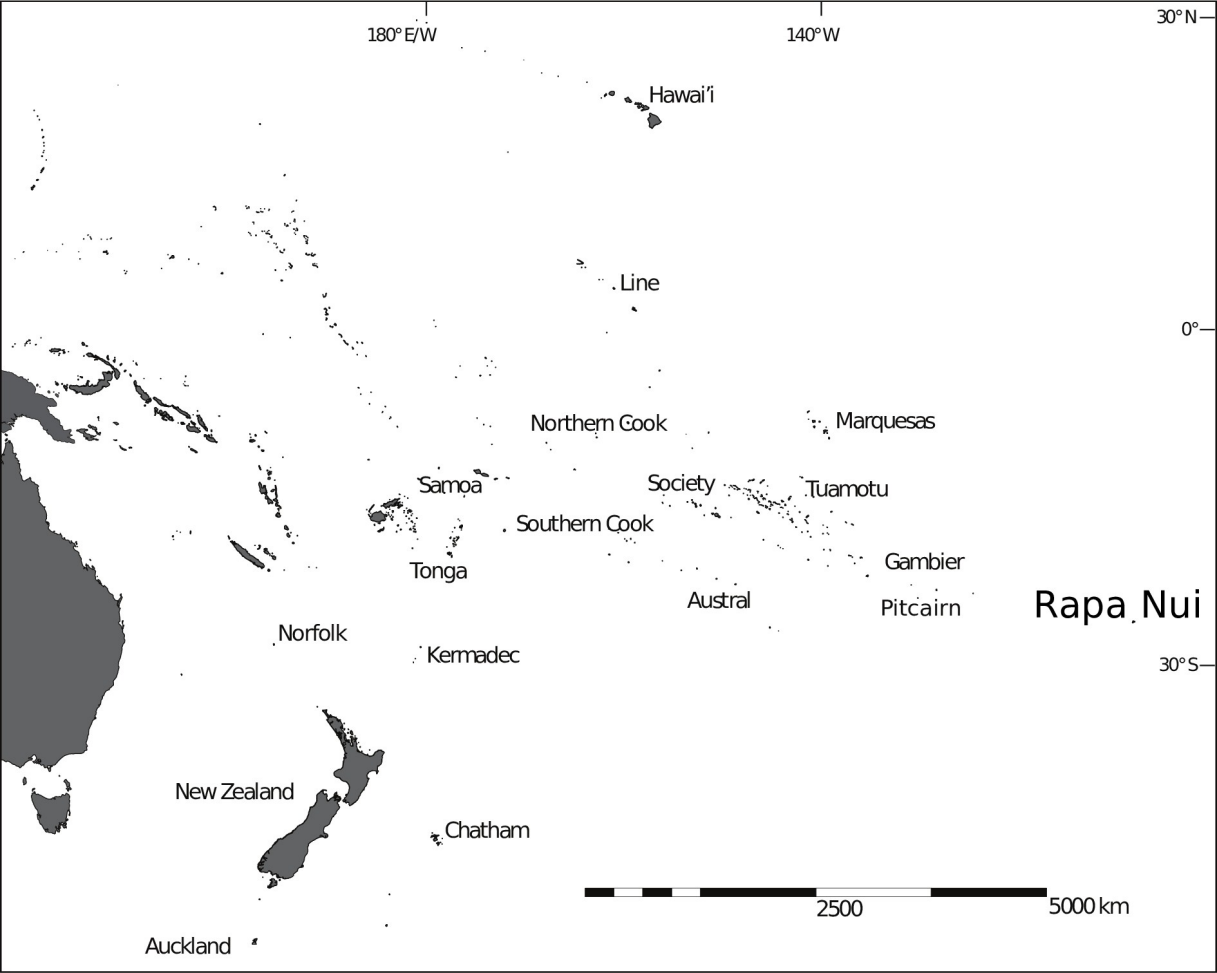
Rapa Nui. The location of Rapa Nui in the easternmost corner of Polynesia.

Rapa Nui is a small (164 km^2^) and remote island in the easternmost South Pacific (Fig 1) that was colonized by Polynesian voyagers in the 12th-13th Century AD [1–4]. The island is perhaps best known for the hundreds of multi-ton stone statues (*moai*) that the islanders constructed and transported over volcanic terrain to every part of the island and placed atop massive stone platforms (*ahu*) [5–7]. The magnitude of Rapa Nui’s monumental architecture is often seen as paradoxical in contrast with the island’s size. At just 23 km in its longest dimension, the island can be easily traversed at a walking pace in under a day. Curiously, for an island so small archaeological and ethnohistoric evidence indicates that pre-contact communities did not interact in an island-wide, panmictic fashion, but rather were organized into a series of distinctive subgroups. Traditionally, at least eight clan groups are known to have spatial territories in the late 19th and early 20th century [8,9]. Detailed analyses of the archaeological record, however, show that many aspects of community interaction likely occurred on an even more localized basis with communities consisting of multiple small, dispersed, and relatively autonomous groups [e.g., 10,11]. Analyses of spatial variability in biological and cultural traits suggest an island population structured into many relatively independent communities organized around *ahu* locations and their immediate local resources [e.g., 12,13]. This evidence points to a significant degree of diversity over small geographic areas on the island in cultural and biological traits. While this diversity and degree of localized community structure is unexpected for such a small island, no model-based hypotheses have been presented to account for these patterns.

Models of random drift offer a productive source of hypotheses for the patterns and distribution of diversity. Drift, whether among inherited genetic or cultural variants, is the change produced by the underlying mechanisms through which variation is sampled and passed on through time. Understanding how random drift can shape cultural diversity has been a significant focus of investigations for cultural evolutionary studies [e.g., 14–19]. Using this concept, researchers have explored how one can detect drift from other factors that shape changes in trait frequencies through time and space [e.g., 20–27]. As has been demonstrated, the effects of drift depend on the size of the population. In large populations, drift tends to cause relatively small changes in trait frequency. In small populations, however, drift can lead to rapid changes in trait frequencies, potentially resulting in fixation or loss of variants. In these situations, drift can produce dramatic differences in historic outcomes: two populations drawn from a single population may begin with an identical frequency of traits, but can rapidly diverge in composition. As we explore in this paper, the effect of drift in small populations often leads to rapid loss of richness (i.e., the number of variants within a population) and diversity (i.e., the distribution of variants within a population) [28].

The factors that drive the effects of drift—population size in particular—have received a great deal of attention as a possible explanation for cultural change. On one hand, some have argued that demography and population sizes strongly shaped cultural history. For example, Henrich [29] argues that the loss of technology among Tasmanian communities throughout the Holocene was a consequence of drift acting on the reduced size of isolated populations. In an opposite scenario, Powell et al. [30] have argued that increases in population sizes were part of the process leading to the explosion of technology and cultural variants in the Late Pleistocene [see also 31–33]. Yet, these kinds of explanations have been strongly criticized [e.g., 34–41].

While debate continues about whether changes in population size explain particular cases of cultural variability, it is well-established that drift contributes to cultural change among small and isolated populations [e.g., 31,42–47]. Population size, however, is not the only significant factor that can change the degree to which drift leads to changes in cultural variability. Of particular note is the work of Premo [24,25,48,49], who has demonstrated how population structure, in particular, plays a key role in shaping diversity in populations. Derex and colleagues [50–52] have also highlighted how the degree of population fragmentation maximizes the rate of accumulation of cultural traits. In particular, partially connected populations can produce highly diverse solutions to adaptive problems not possible in fully connected groups [50].

Understanding the effect of drift on cultural variability is particularly important in the study of island settings where populations tend to be isolated to relative degrees and limited in size compared with continental contexts. Small and isolated island populations provide model systems to study the relationship of limited population size, drift, community patterning, and interaction on cultural diversity [e.g., 12,53–55]. For islands, the key aspect of drift and its effects is not just on factors that favor cultural trait accumulation [50,56,57], but also those favoring cultural trait *retention* [51,52]. The retention of traits can be beneficial when they confer information about past states and cumulative cultural solutions to environmental and social problems [58] (p.278). In isolated locations such as islands and where populations regularly face uncertainty about future environmental conditions [e.g., 59–63], cumulative cultural knowledge has great potential value. Thus, mechanisms and strategies that preserve diversity and richness likely have fitness consequences. To examine factors that favor trait retention, we present forward time simulations that permit us to configure populations and their interactions in various ways. We first establish that the simulations conform to the expectations of the drift model and the findings of previous researchers [e.g., 24,28,50,52]. We then use this model to develop hypotheses about the history and community structure of Rapa Nui (Fig 1). Using the cultural transmission models presented here, we hypothesize that the observed patterning of community interaction may be related to the maintenance of cumulative cultural information. We then review archaeological spatial data from Rapa Nui to evaluate community patterns within the context of this model. While some components of the model predictions are difficult to thoroughly test given the somewhat limited archaeological data, the overall patterns in biological and artifactual remains suggest broad agreement with the core model expectations. We conclude with a discussion of these limitations as areas for future research.

## Networks, population structure, and drift

The effects of random drift on variability are mechanical and largely depend on population sizes. Yet a significant factor is variability in population structure, which can play a key role in shaping cumulative cultural diversity in populations [e.g., 24,25,48–52]. From these studies, it is clear that the impact of drift is greatest when populations are well-mixed. Or conversely, the greater the degree of structure within a population, the more likely that variability will be retained, all other things held constant. The relation between population structure and drift can be demonstrated by modeling population interaction as a network. Structure within a population can be represented by a network where vertices represent individuals (*N*) and edges represent the potential interaction between those individuals (e.g., mating or social learning). The structure of the network then varies by the number of edges between individual vertices (*k*, the network degree), from immediate neighbors (*k* = 2) to all other vertices (*k* = *N*-1).

As Schneider and colleagues [28] have shown, the effects of drift on diversity can be countered by a combination of mutation (or innovation) rate and/or highly structured (low *k*) networks. Following Schneider et al. [28], given a population of size N and mutation rate μ, drift dominates whenever 2*μN*⪡1. For 2*μN*⪢1, on the other hand, mutation dominates over the effect of random drift, maximizing diversity. The transition occurs at a threshold, *μc* = 1/2*N*, where the equilibrium distribution of trait frequencies becomes uniform. This threshold (*k*_*c*_) is a critical point, above which random drift is insensitive to population spatial structure. Below *k*_*c*_, small degrees of connectivity (*k*) offer high degrees of spatial structure that, when combined with mutation rate, lead to increases in overall diversity [e.g., 64,65]. In this way, the diversity of traits can be increased either by increasing the mutation rate at fixed *k* or by decreasing *k* at a fixed mutation rate. Schneider et al. [28] (p.15) remark that “in consonance with classical results, extreme restriction in [gene] flow is required for structuring to affect. In fact, the mutation rate above which drift is overcome changes significantly only when the degree of the network becomes very small.”

In terms of information retention within a population, our interest is not focused on the effect of mutation (or innovation), but on population structure (*k*). While innovation serves to increase overall diversity, its effect is to alter that information and thus the potential contribution it might have to future fitness consequences. Population structure, on the other hand, reflects the way individuals or groups of individuals within a population interact and could serve to retain information. To explore this effect, we need to model various consequences of different configurations of populations. Given the aggregate nature of the archaeological record, these models accommodate populations interacting at two different scales: individuals interacting within a small community of well-mixed structure, and those communities interacting amongst one another.

To illustrate how population structure affects diversity and the retention of traits, we used simuPOP [66,67] to simulate drift within populations of varying configurations. SimuPOP is a Python-based population simulator that allows one to evolve populations forward in time under varying configurations of mutation, recombination, migration, and population/subpopulation sizes. We based our simulations on a simple Wright-Fisher model [68,69] that explores changes in a haploid population of fixed size, *N*. Traits are modeled as values within a single locus, in a way that is equivalent to attributes along a single dimension [sensu 70]. In the model, traits for individuals are derived from each time-step by sampling with replacement from the pool of other individuals (i.e., the previous “generation”). This pattern of copying traits is effectively random, implying that an individual has an equal probability to interact with anyone else in the population. At each step when an individual copies traits, there is a fixed chance of innovation where a new trait is introduced.

**Fig 2:**
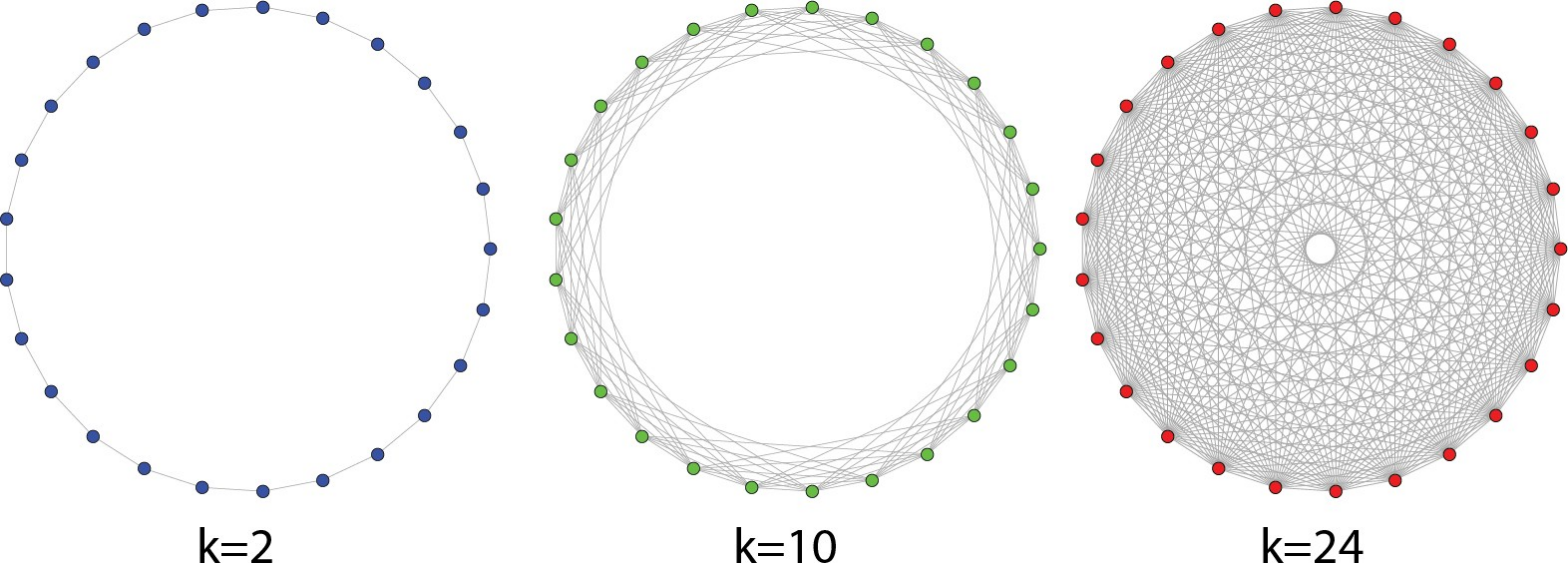
An example of three network configurations of 25 subpopulations connected to varying degrees. These networks are Watts-Strogatz small-world graphs [71]. The colored circles represent individual subpopulations and the lines between the circles represent the interaction between subpopulations. This figure shows three configurations of subpopulations connected just to neighbors (*k* = 2) to 10 other subpopulations (*k* = 10), and all other subpopulations (*k* = 24). In these models, there is an equal probability of copying between all connected subpopulations.

To model variation in population structure, we divided the overall population into a varying number of subpopulations. Within each subpopulation, copying is assumed to be random but is not allowed between subpopulations. With subpopulations, drift can produce a unique combination of traits even if the initial conditions begin identically. Subpopulations can be configured to interact with other subpopulations by copying traits depending on number and pattern of links between points in the configuration (Fig 2). In simuPOP, we can also vary the between-group interaction rate (modeled in SimuPOP as “migraption probability”), the likelihood that individuals from a subpopulation copies traits from other subpopulations. All code for these simulations is available at https://github.com/clipo/network-drift.

Our simulations explore the impact of 5000 individuals separated into a series of subpopulations that interacted under varying configurations (S1 Table). Our simulations are designed to approximate a single population on an island organized into a series of interacting groups (subpopulations); a pattern evident on pre-contact Rapa Nui [e.g., 10,11,72]. To isolate the effects of drift, we set the innovation rate to zero and began with identical subpopulations with an even distribution of traits along a single locus. In the simulation, each timestep is composed of an event where individuals in subpopulations copy traits randomly from within their subpopulation, and with a small probability of copying traits from another connected subpopulation. Note that these timesteps are not meant to represent “biological” generations, but simply repeated steps of maximum social learning among all individuals. To calculate a 95% confidence interval for diversity measures of the population over time, we aggregated values from multiple runs (e.g., 10 or more).

To evaluate the effects of network structure on diversity, at each time step we calculate diversity in trait frequencies in subpopulations using an F_ST_ statistic [73], a widely used estimator of Wright’s fixation index. The F_ST_ statistic is a measure of population differentiation based on trait differences between populations. F_ST_ is calculated as the correlation of randomly chosen trait values within the same subpopulation relative to that found in the entire population. F_ST_ is calculated as:

$$F_{ST}=\frac{\sigma_{S}^{2}}{p(1-p)}$$ (Eq 1)

where *p* is the average frequency of the trait in the total population, **σ**_S_^2^ is the variance of the frequency of the trait between different subpopulations. Values for F_ST_ range from 0 to 1. F_ST_ should be close to zero if populations are identical in their trait distributions. Values of 1 indicate significant differentiation among populations.

Schneider et al. [28] demonstrate that variability in network connectivity between individuals will affect overall diversity. For Rapa Nui, we were interested in examining whether community interaction patterns would promote the retention of information over time, thus providing a potential benefit to communities under changing and unpredictable conditions. To assess this possibility, we look at diversity, the distribution of traits in the population, and richness, the number of traits in the population. Thus, in our simulation, we measure F_ST_ as well as track richness values of traits in the overall population over time to see how network structure affects these values.

### Network configuration

**Fig 3:**
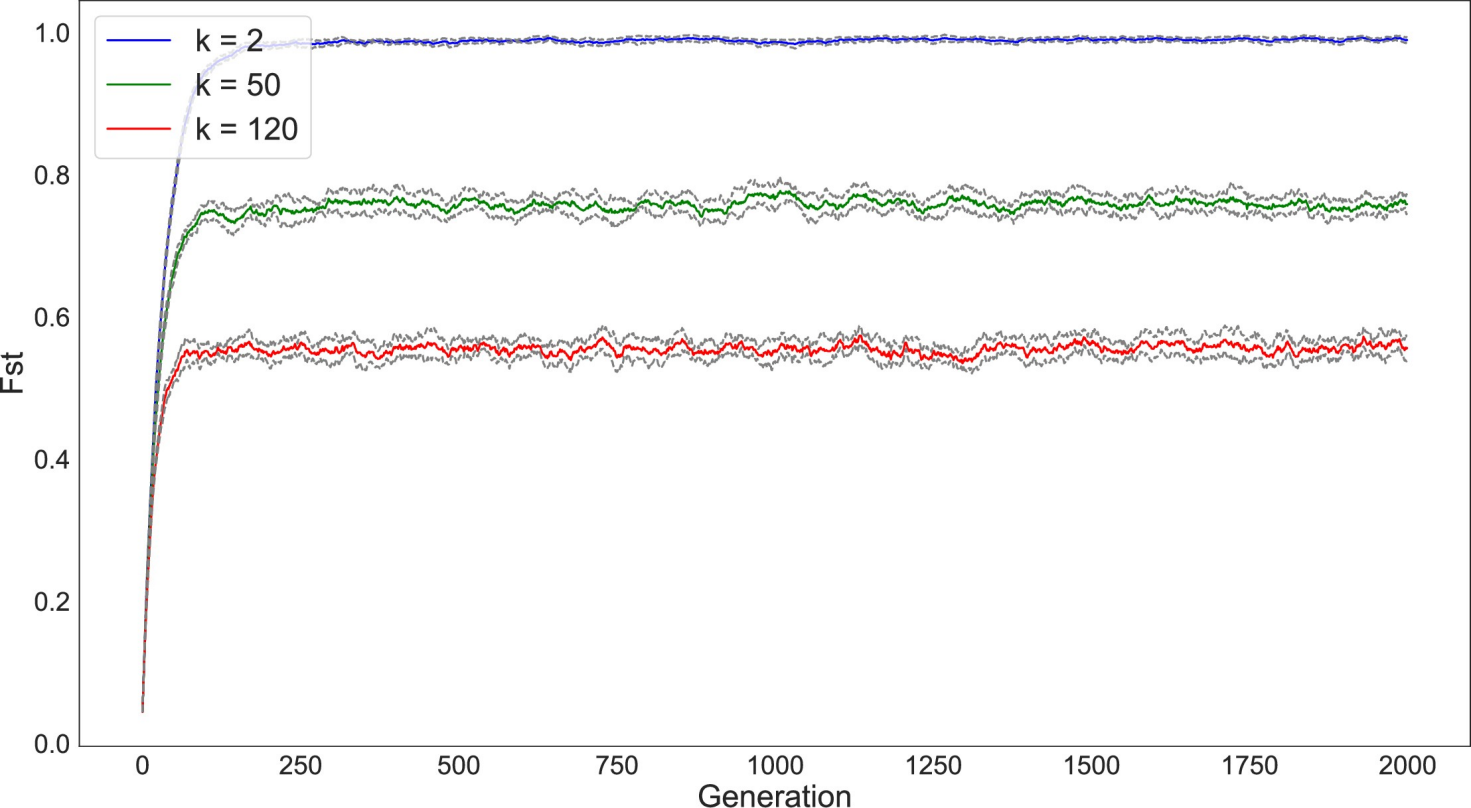
Change in diversity (as measured by F_ST_) over 2000 timesteps of a simulation with 200 subpopulations under varying network connectivity conditions (*k* = 2 [blue], *k* = 50 [green], *k* = 120 [red]). Subpopulations are initialized with an even distribution of traits. Due to drift, diversity increases the greatest in the set of subpopulations that have the least degree of connectivity. In this and following figures, the grey bands around each data series represent the 95% confidence interval of the statistic across all simulation runs.

**Fig 4:**
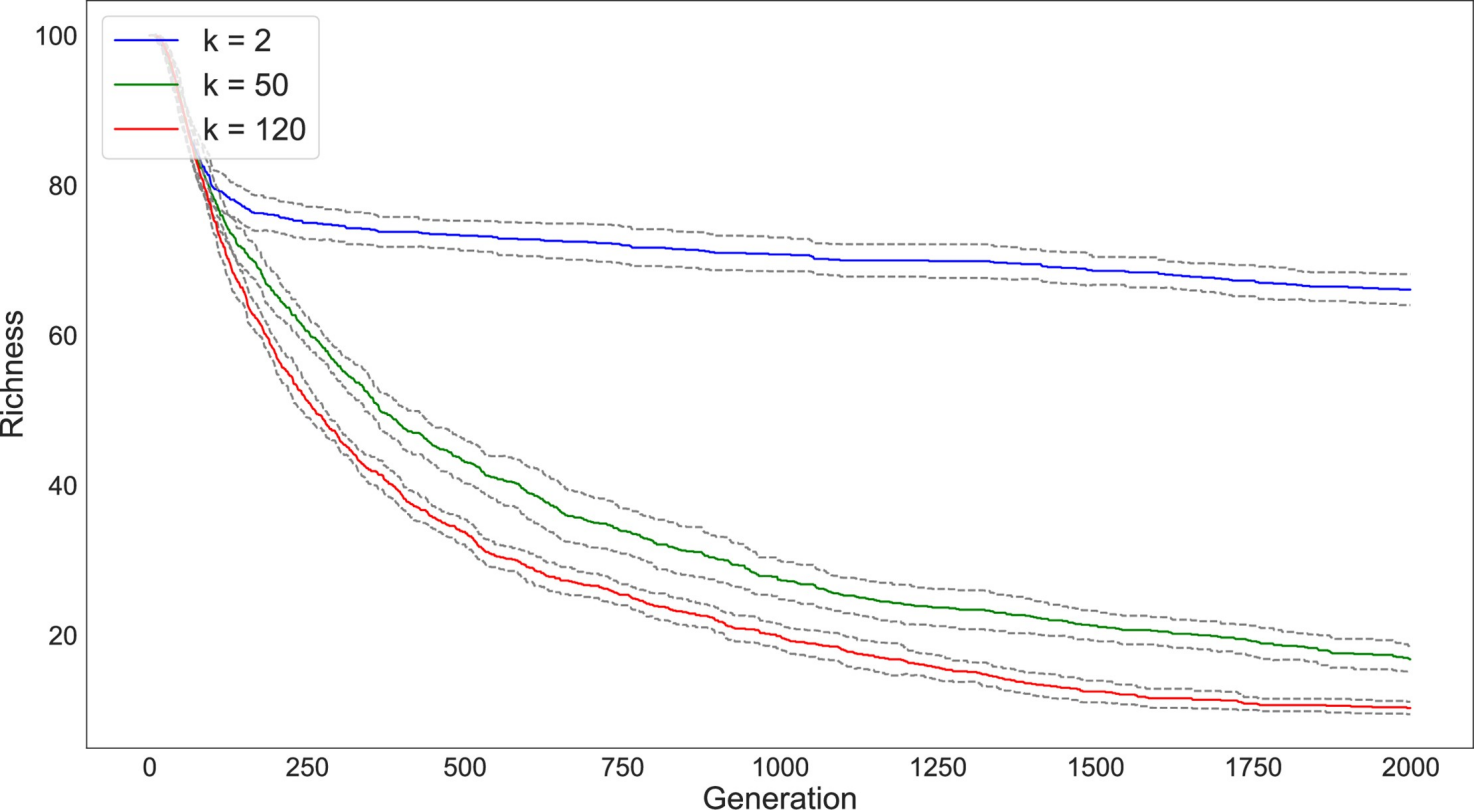
Change in richness (number of different traits present in the population) over 2000 timesteps of a simulation with 200 subpopulations under varying network connectivity conditions (*k* = 2 [blue], *k* = 50 [green], *k* = 120 [red]). In these simulations, populations begin with the same degree of richness. The loss of traits is dramatically quicker for networks with a greater degree of connectivity.

We first simulated 5000 individuals divided into 200 subpopulations and varied the number of connections between the subpopulations from *k* = 2, *k* = 50, and *k* = 120 (Figs 3 and 4). We ran each simulation for 2000 timesteps and tracked diversity and richness measures for each run. Multiple runs of the simulation provide a 95% confidence interval for the diversity and richness values and are represented as the grey lines surrounding each statistic in the following graphs. As seen in the figures, our results match the expectations of Schneider et al. [28]: the magnitude of drift’s effect on variability depends on the degree of network connectivity. The populations that are split into subpopulations with the lowest degree of connectivity (e.g., *k* = 2) retain the greatest diversity and richness compared to those configurations with higher degrees of connectivity.

### Network distance

The connectivity between vertices is just one dimension of how spatial configurations of populations can alter the effect of drift. The more general component of network structure is the overall distance between the vertices. The greater the distance between vertices (subpopulations), the lower the overall effects of drift. Distance between vertices is determined by two factors. First, the number of edges between vertices contribute to distances—the more edges that connect to each vertex, the shorter the distance and the greater the influence of drift on the population overall. Second, we can vary the number of vertices (i.e., subpopulations) that make up the population—the greater the number of vertices, the larger the distance between any two locations given the network connectivity. Thus, populations divided into an array of smaller subpopulations will experience the effects of drift less than those that are divided into fewer, larger subpopulations.

**Fig 5:**
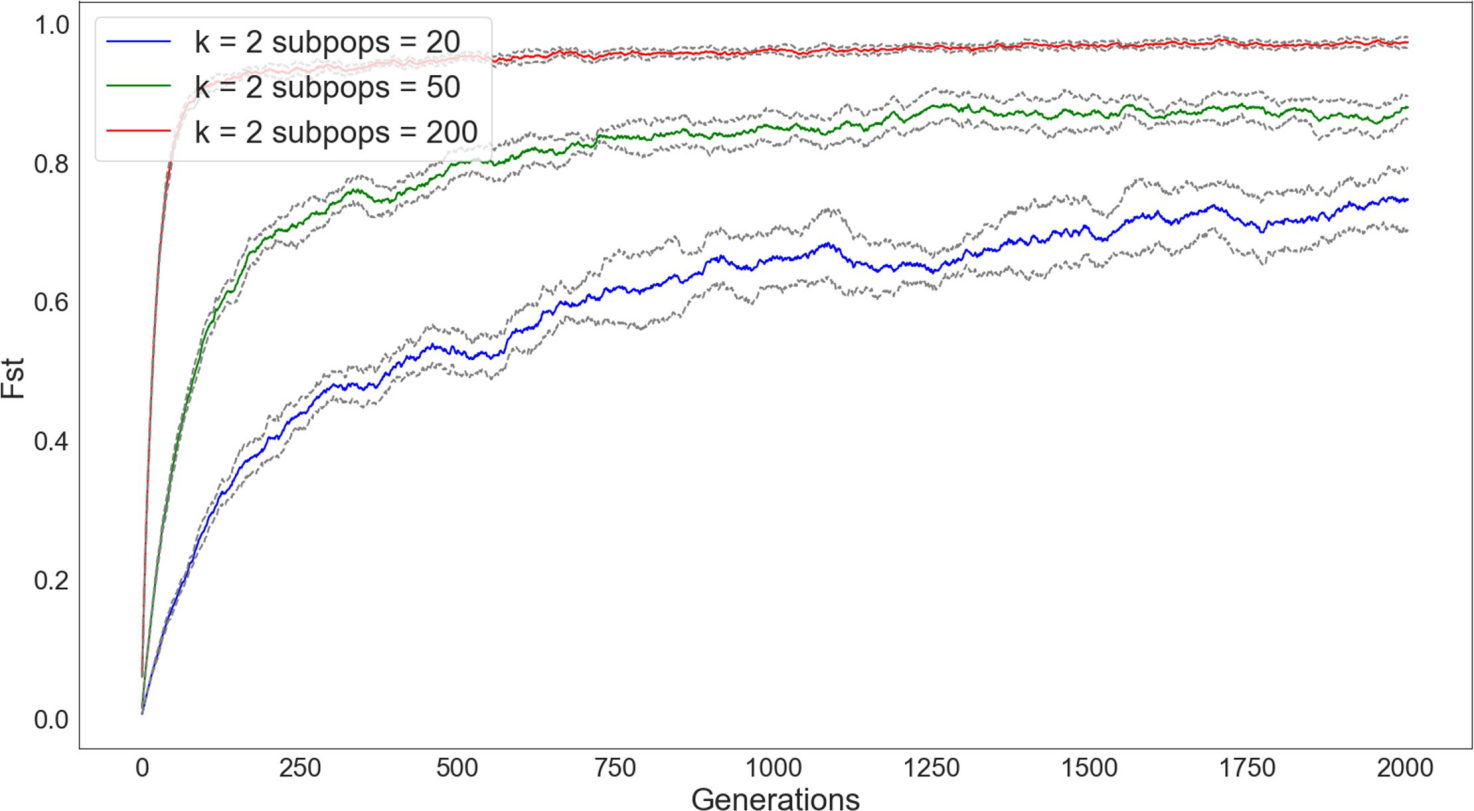
Changes in diversity over 5000 timesteps of a simulation in which the network connectivity is kept constant (*k* = 2) but the number of subpopulations is increased (subpopulations = 20 [blue], subpopulations = 50 [green], subpopulations = 200 [red]). In these simulations, populations begin an even distribution of traits. Diversity is substantially higher for network configurations with a larger number of subpopulations.

**Fig 6:**
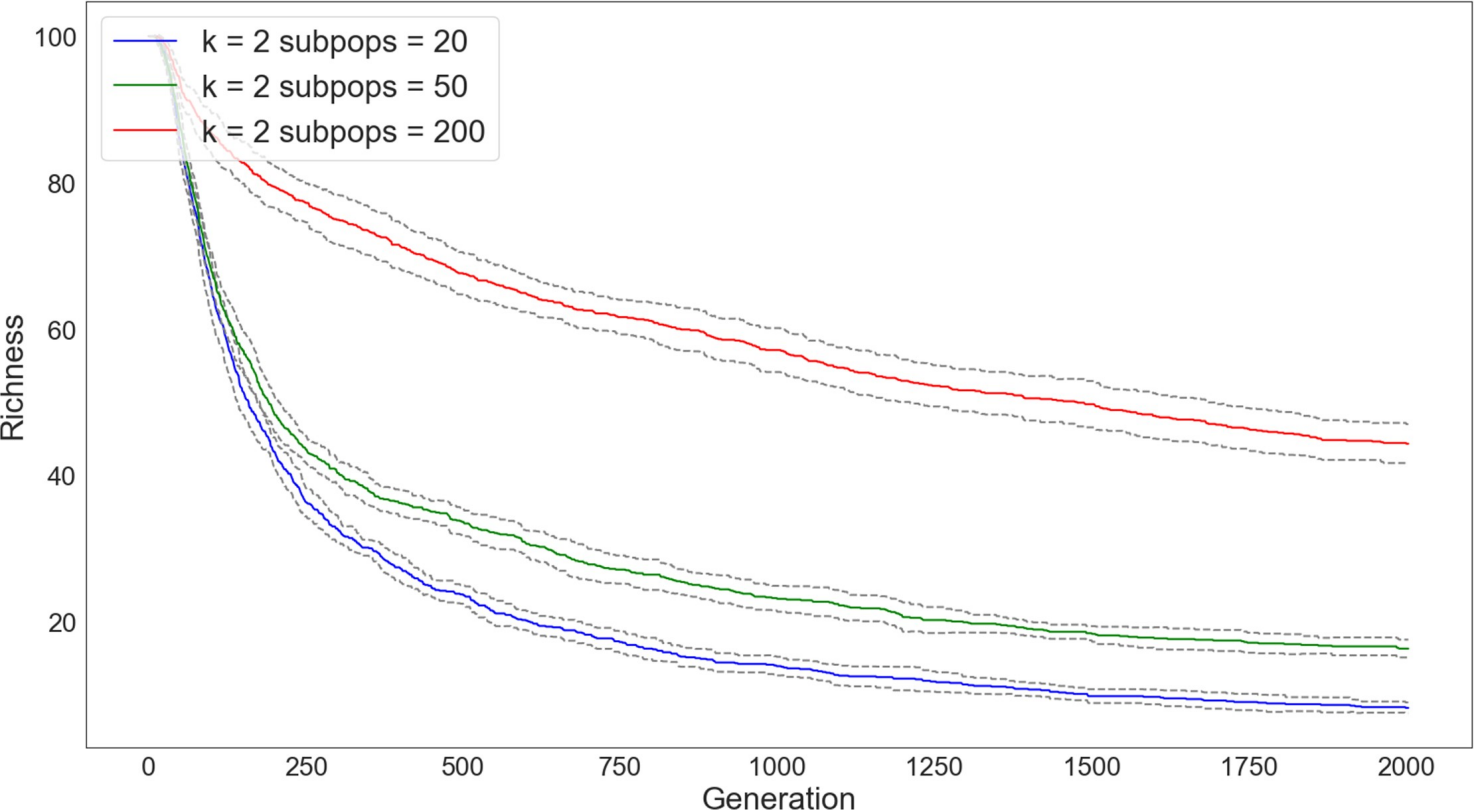
Changes in richness over 5000 timesteps of a simulation with network connectivity kept constant (*k* = 2), but the number of subpopulations increased (subpopulations = 20 [blue], subpopulations = 50 [green], subpopulations = 200 [red]). In these simulations, populations begin with the same degree of richness. The loss of traits is dramatically quicker for networks with a fewer number of subpopulations.

We demonstrate this effect in a series of simulations varying the number of subpopulations (Figs 5 and 6). Starting with a fixed population size of 5000, we vary the number of subpopulations by 20, 50, and 200, but fix the connectivity between subpopulations to just neighbors (i.e., *k* = 2). We then track diversity and richness over 5000 timesteps. As in the case where we varied the degree of connectivity, the populations divided into more subpopulations led to greater diversity and richness, even when subject to the same degree of connectivity.

The key finding is that the most critical factor in preserving variability in a population is the *graph degree*: the number of vertices between any two edges. The higher the graph degree, the greater the potential retention of diversity and richness. In a population structure modeled as a graph, degree increases inversely with the number of interactions between subpopulations as well as with the number of subpopulations. Given this factor, we expect population structures that are modeled as large small-world graphs [71], where subpopulations are linked only to neighbors, as particularly effective in retaining diversity and richness. In contrast, populations structured as random graphs with a low degree as well as those with hierarchical structure will tend to be less effective in retaining diversity and richness as a result of drift.

### Interaction rate between communities

**Fig 7:**
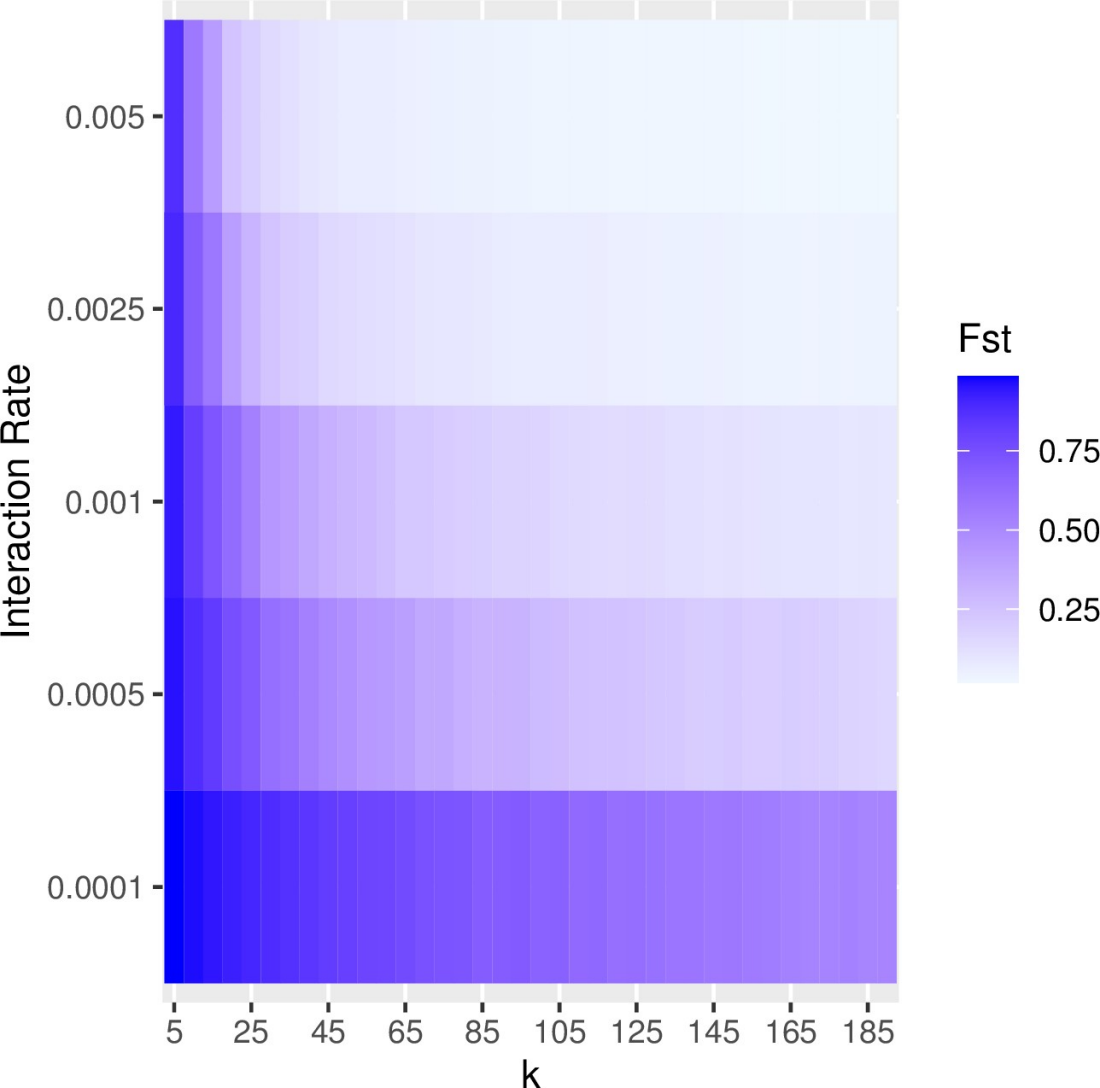
The effects of network structure and the rate of between group-interaction on overall trait diversity within a population. In this set of simulations, we divided populations of 5000 into a series of 200 subpopulations and varied connectivity from *k* = 5 to *k* = 190 in steps of 5. For each value of *k*, we ran the simulation with between-group interaction rates that ranged from 0.0001 to 0.005. The resulting data are the mean values of F_ST_ calculated from 10 runs at the point of 2000 timesteps. The values of diversity are shown as a heatmap where the high levels of diversity are illustrated in dark blue and the low levels are in light blue. Diversity is best maintained under conditions of the low rate of between-group interaction and low levels of connectivity.

**Fig 8:**
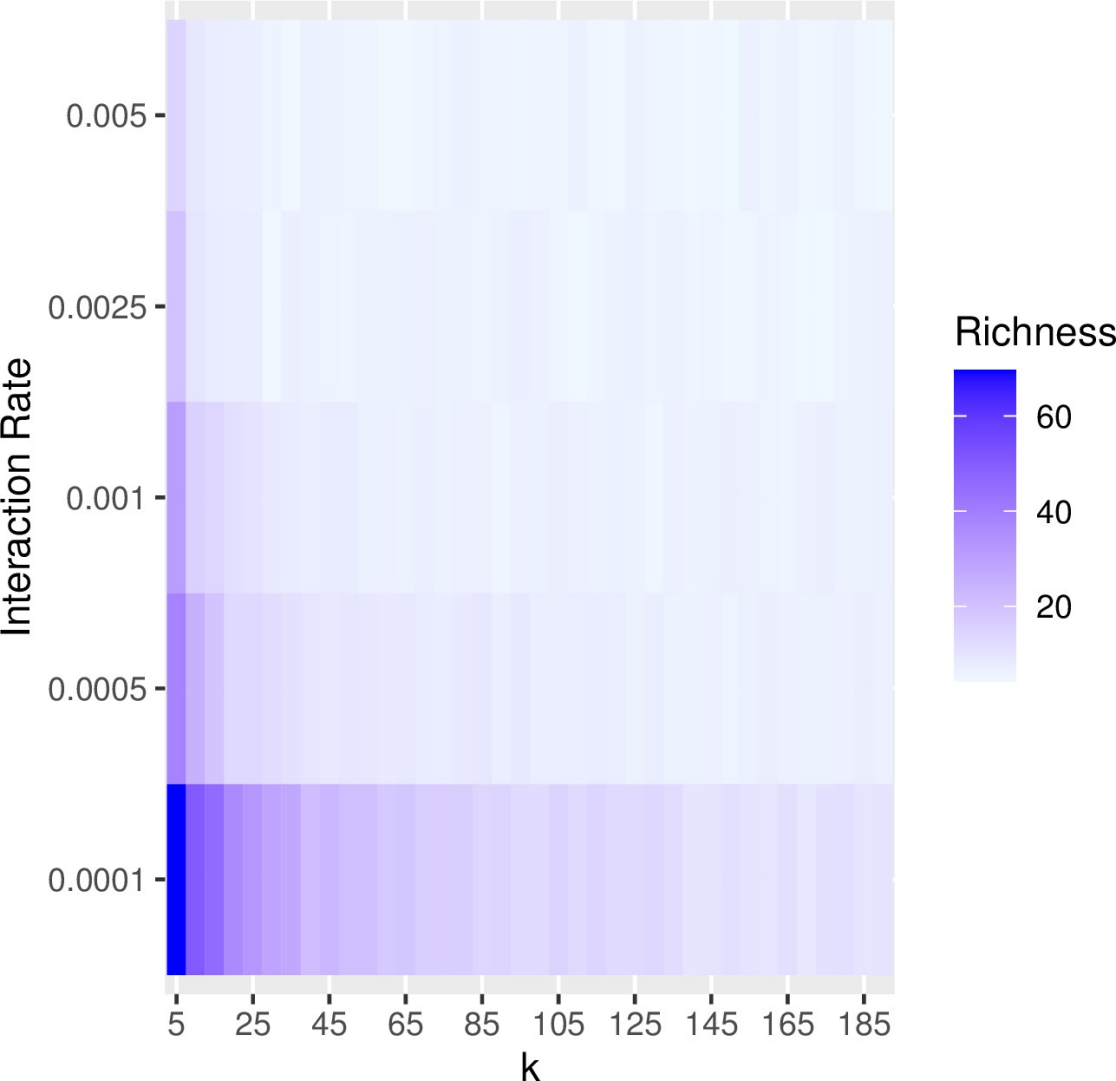
The effects of trait network structure and between-group interaction rates on trait richness within a population. In this set of simulations, we divided populations of 5000 into a series of 200 subpopulations and varied connectivity from *k* = 5 to *k* = 190 in steps of 5. For each value of *k*, we ran the simulation with the rate of between-group interaction ranging from 0.0001 to 0.005. The resulting data are the mean values of richness calculated from 10 runs at the point of 2000 timesteps. The richness of traits is best maintained under conditions of relatively low rates of between-group interaction and low levels of *k*.

**Fig 9:**
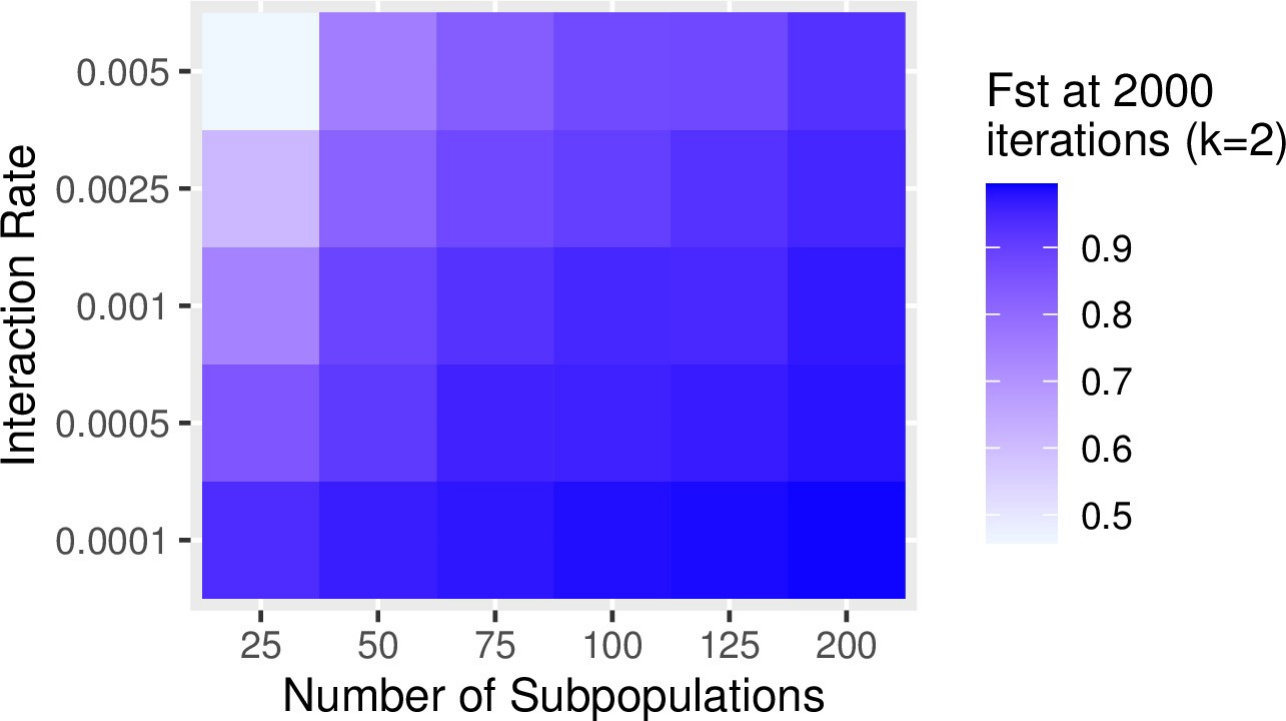
Diversity measured by F_ST_ among a population at 2000 timesteps with network connectivity of *k* = 2 with a variable number of subpopulations (5–200) and between-group interaction rates (0.0001–0.005).

**Fig 10:**
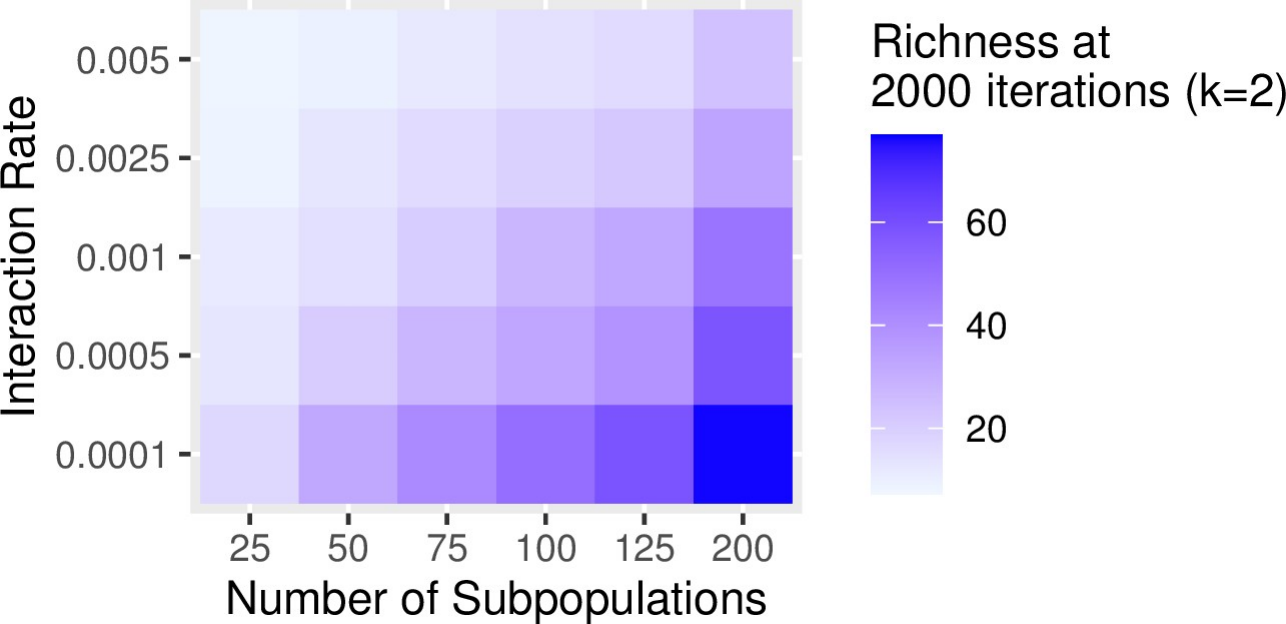
The richness of traits in a population at 2000 timesteps with network connectivity of *k* = 2 with a variable number of subpopulations (5–200) and between-group interaction rates (0.0001–0.005).

In the previous examples, we set the rate of interaction between communities (i.e., the probability of copying between subpopulations, referred to as the “migration rate” in the SimuPOP simulation package) to constant at a low level (i.e., 0.25%/timestep). Increasing the probability of interaction has a similar effect of increasing the impact of drift on population diversity and richness. The greater the migration probability, the greater the effects of drift. Figs 7 and 8 show the results of a series of simulation runs in which we varied the probability of between-group interaction (from 0.0001 to 0.005) and the degree of connectivity (from k = 5 to k = 190) while keeping the number of subpopulations constant (N = 200). The values of the between-group interaction rates are arbitrarily chosen to be low relative to the number of individuals in each subpopulation. The choice of the number of subpopulations evaluated is also arbitrary and selected to evaluate a range of at least one order of magnitude. The combination of low between-group interaction probability and low connectivity between subpopulations is especially potent in retaining richness. This pattern is consistent with our findings on how the number of subpopulations and rate of between-group interaction influence the richness and diversity: significant network degree results in greater diversity and richness (Figs 9 and 10).

### The retention of rare traits

Our simulation results demonstrate the expected effects of drift as presented by Schneider et al. [28]: the degree of network distance and reduced interaction among subsets of individuals in a population strongly influences the retention of traits that would otherwise be lost to drift. In remote and isolated island settings, loss of traits could have potential implications for survival. The retention of traits would have particular value in the context of uncertainty or facing problems that occur only rarely. If traits offer information about past conditions, then they might offer advantages when similar conditions return. At the scale of an island population, only one group on the island would need to retain traits and share and act on those traits at an appropriate time. This condition would likely contribute to group-level fitness differences through differential sorting of community behavior. Thus, finding the conditions that maximize the retention of rare traits potentially sheds light on ways island communities adapted to serve this purpose.

**Fig 11:**
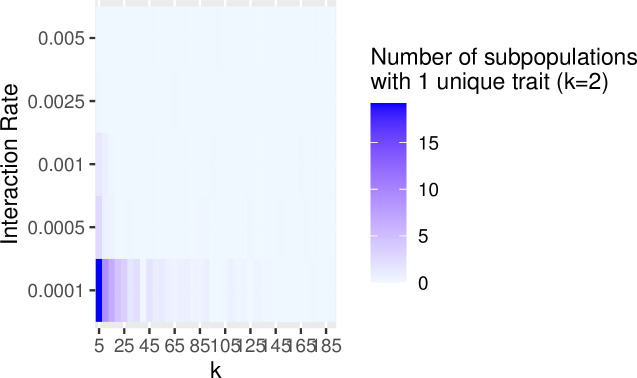
The impact of network structure is determined by connectivity and rates of between-group interaction on the number of traits found only in one subpopulation after 2000 timesteps.

**Fig 12:**
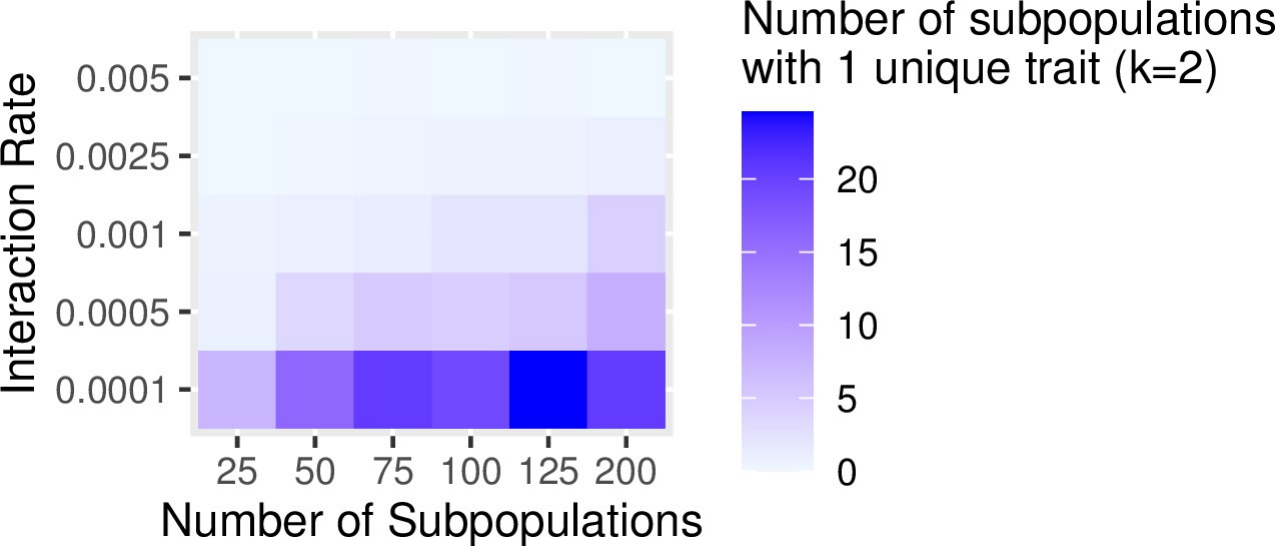
The impact of network structure is determined by the number of subpopulations (*k* = 2) and the rates of between-group interaction on the number of traits found only in one subpopulation after 2000 timesteps.

Our simulations illustrated these conditions by exploring the configurations of the between-group interaction rate and degree of connectivity that favor the retention of rare information by examining the number of subpopulations that hold a unique trait not shared with any other subpopulation. For each combination of *k* and rate of between-group interaction, we ran the simulation for 2000 timesteps and then counted the number of subpopulations that held a trait not found elsewhere. As expected, the combination of factors that favor retention of rare traits consists of low between-group interaction and low levels of connectivity (Figs 11 and 12). Population structure, therefore, plays a key role in preserving rare but potentially crucial information.

## A model for population structure on Rapa Nui

Located almost 2000 km from Pitcairn Island, its closest intermittently inhabited neighbor, the island is one of the most isolated places on Earth. The archaeological record suggests the population was isolated soon after initial settlement, with little, if any, interaction with other islands of Polynesia [3,72,74,75]. The island’s subtropical environment is also quite marginal, with poor soil nutrients, limited surface freshwater, and no large coral reefs or a lagoon. While the island never had abundant resources or rich soils, it was transformed by humans over ca. 500 years through the introduction of the commensal Pacific rat [76–78], forest clearance [79,80], and the establishment of vast lithic mulch gardens for food production [e.g., 81–87]. Recent studies show that freshwater sources available in groundwater discharge (springs) predict the locations of *ahu* and point to community activities centered on this shared critical resource [88–90]. Given the diminutive size of Rapa Nui and its relatively marginal environment, the island never supported a particularly large population. While there have been claims of large population sizes on the order of 17,500–30,000 individuals [e.g., 91–94], the archaeological and historical evidence indicates a substantially smaller population likely around 5,000 maximum [3,8,10,11,72,95].

Unpredictability in rainfall brought critical uncertainty to agricultural productivity and the availability of drinking water [11,12,96]. Based on an analysis of soil, historic rainfall data, topography, and substrate age, Morrison [11] (p.184) shows that “between one time and two times a decade many of the areas of the island are only marginally suitable for agriculture or not suitable at all.” With such unpredictability in conditions necessary for survival (i.e., food and water), past knowledge about problems and solutions would have selective value. For example, if an individual knew how to survive an extended drought using particular cultivation or water management strategies, such as many unique strategies used by Rapanui people [e.g., 87,89], that individual and their community would be better off than those without such knowledge. The effects of drift on this small and isolated population would thus prove challenging for retaining cultural knowledge shared in oral traditions by individual-to-individual social learning mechanisms. Yet, given that the Rapanui people thrived for 500 years before the arrival of Europeans [1,3,97], islanders must have adapted strategies of community patterning and the use of technology to mitigate the effects of drift.

**Fig 13:**
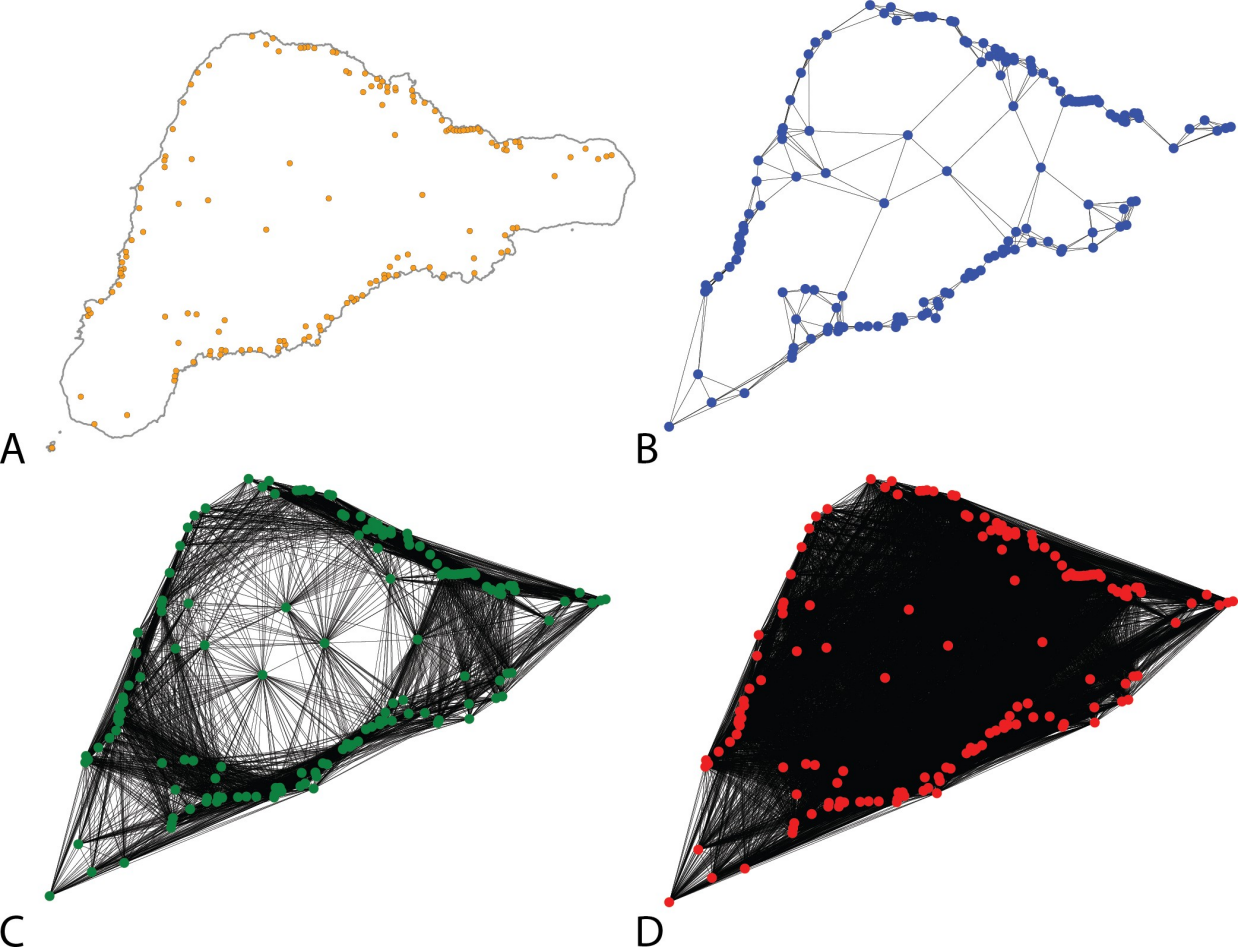
Interaction configurations of Rapa Nui communities as modeled by *ahu* locations. (A) Image *ahu* locations as determined by Martinsson-Wallin [6] and DiNapoli et al. [90]; (B) Connectivity of communities interacting primarily with local neighbors; (C) Population structure of local communities interacting with the 50 nearest other communities; and (D) Nearly panmictic population structure of local communities interacting with 140 other locations.

Based on the effects seen in these simulations, we can examine how the population structure of Rapa Nui could have led to trait retention despite the island’s limited size and isolated, small population. Here, we model various configurations of the population using archaeologically known spatial locations of communities. Following ethnohistoric accounts and archaeological research [e.g., 6,10,11,72], we know that image *ahu* (statue platforms) served as a central feature of numerous small, dispersed communities. We used the locations of image *ahu* to model varying degrees of interaction among these communities and evaluate the outcomes of the overall island diversity and richness of shared traits. The model is parameterized with 150 subpopulations corresponding to the number of *ahu* sites. It is important to note that the number of distinct subpopulations is unknown and we simply use this high number of subpopulations for the purposes of illustration. The key aspect of the model is not the absolute number of subpopulations, but the degree of interaction between them (*k*). Fig 13 shows three configurations of contact between *ahu* communities: from interactions that are limited only to intermediate neighbors (*k* = 5), to a scenario of communities interacting regionally with 50 other locations (*k* = 50), and to a configuration of communities interacting with nearly all other locations (*k* = 140). Using 5000 individuals as the overall population of the island and a low rate of between-group interaction (0.0001), we simulated interaction and tracked diversity and richness at the scale of the island.

**Fig 14:**
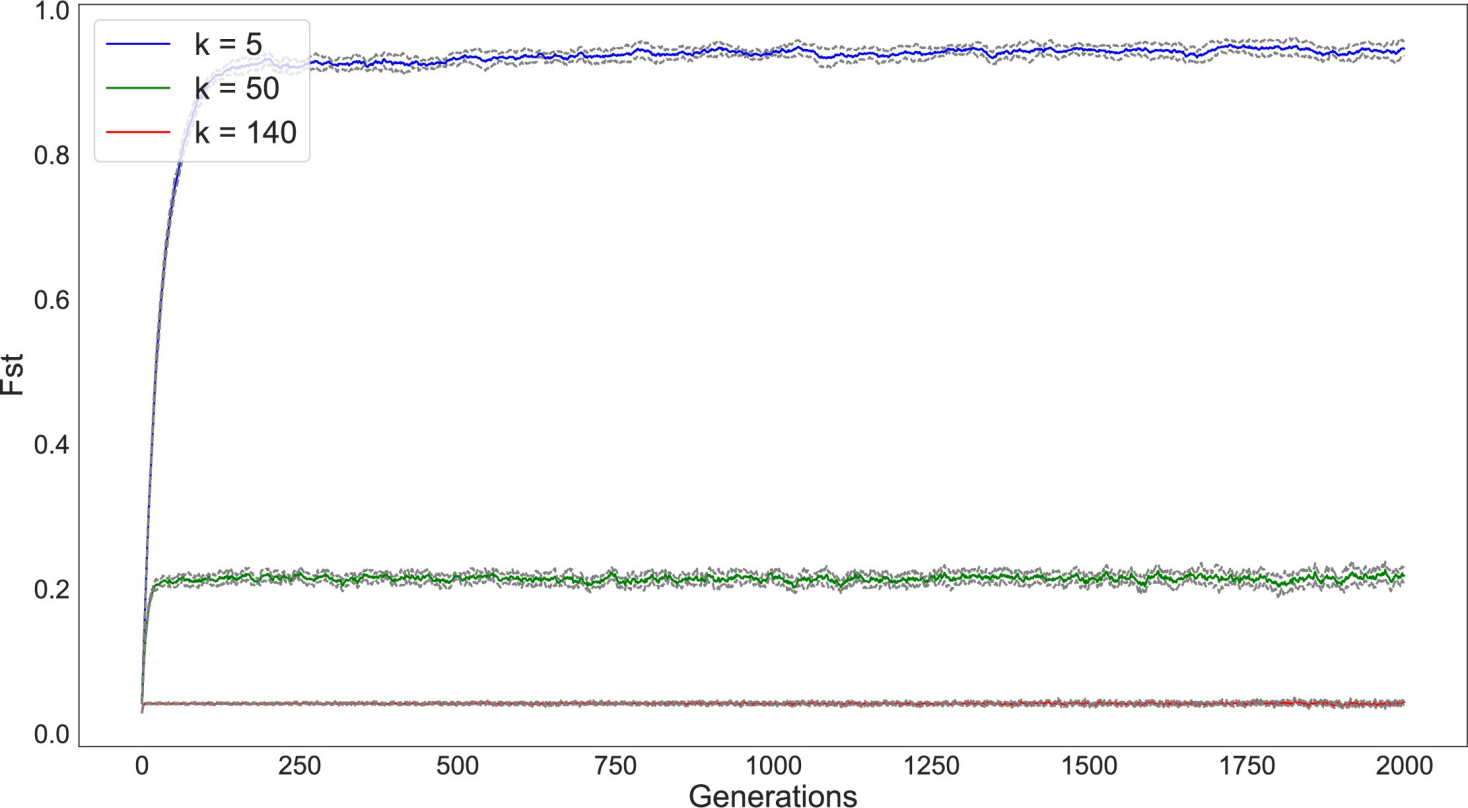
Impact of drift on trait diversity (measured as F_ST_) on Rapa Nui communities located around image *ahu* under three network configurations (*k* = 5, 50, 140).

**Fig 15:**
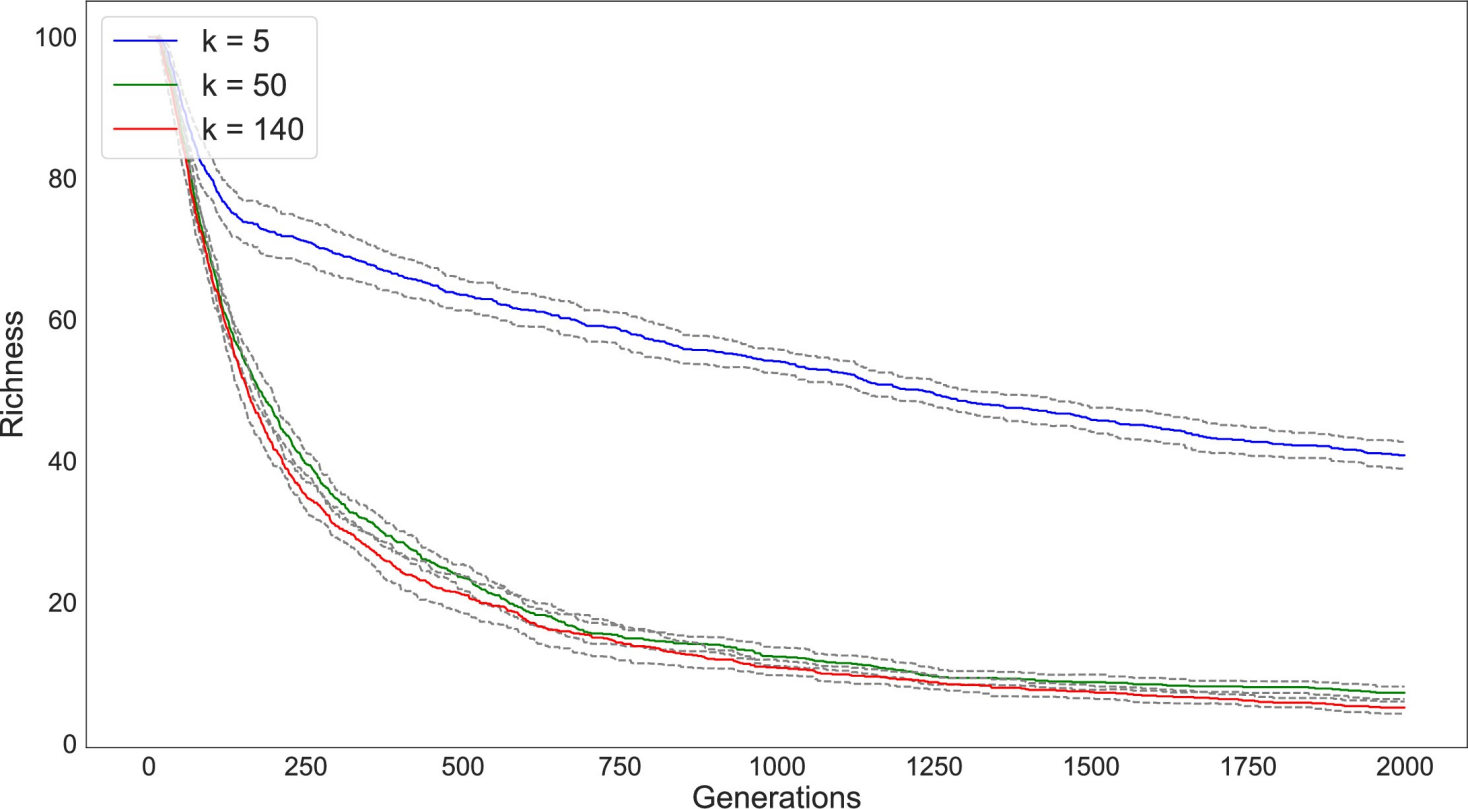
Impact of drift on trait richness on Rapa Nui communities located around image *ahu* under three network configurations (*k* = 2, 50, 140). Notably, the lower the degree of connectivity (*k*) the greater the retention of traits.

**Fig 16:**
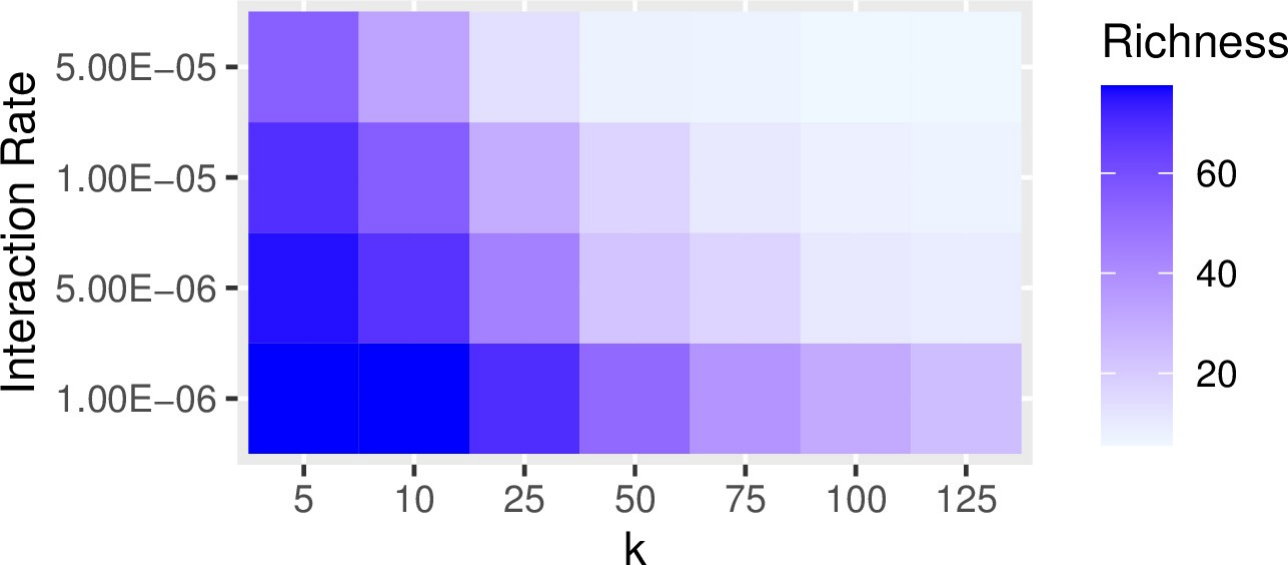
Trait richness after 2000 timesteps in the set of subpopulations modeled on Rapa Nui as a function of network connectivity (*k*) and between-group interaction rate.

**Fig 17:**
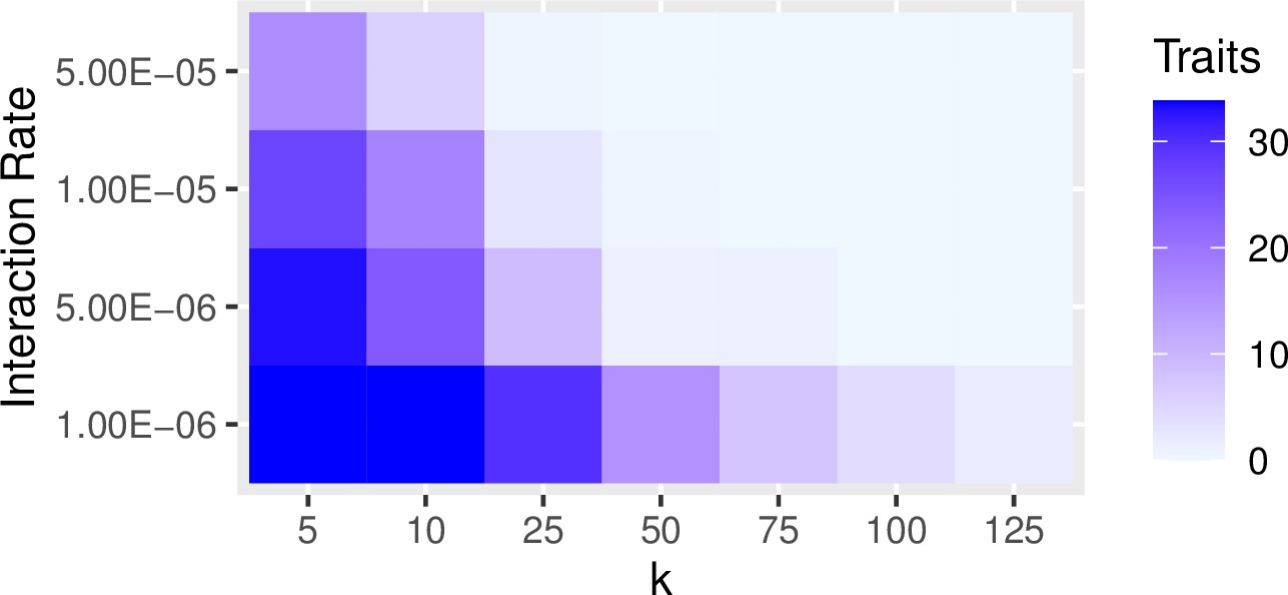
Traits found only in one subpopulation as a consequence of network connectivity (*k*) and between-group interaction rate after 2000 timesteps.

Using these models, we simulated population interaction for 2000 timesteps under conditions of low between-group interaction (i.e., 0.0001/copying event) and with no mutation (Figs 14 and 15). Note that the length of the simulation time steps is somewhat arbitrary and was selected simply to ensure convergence to a steady state. As expected, conditions of extremely low connectivity (*k* = 5) resulted in a high degree of diversity (measured as F_ST_) and retained the greatest degree of richness over the span of the simulation. We also conducted a parameter sweep to look at the relations between between-group interaction rate and *k* network connectivity (Figs 16 and 17).

Overall, our findings suggest that if conditions on Rapa Nui favored the retention of information, we would expect the patterns of interaction among communities to be highly localized—strongly spatially biased to the point that individual communities would primarily share information internally and with limited interaction to just immediate neighboring communities.

### Rapa Nui community patterning

Given the consequences of drift structured by the interaction between subpopulations, do the modeling results help us account for the observed archaeological record of the island? The archaeological evidence suggests the number of subpopulations was high relative to the overall small size of the island. Settlement pattern analyses of the surface archaeological record support the notion that pre-contact communities were remarkably small, numerous, and centered around *ahu* locations [10,11,72]. While we lack comprehensive chronological information for many *ahu*, a recent model-based synthesis of radiocarbon dates indicates that at least some of these ceremonial platforms were built and used contemporaneously over the course of the island’s history [1]. While Stevenson [98–100] argues that there were just 11 communities on the island, this claim is founded on the assumption that the largest 11 *ahu* structures were the focal points for the larger territorial units. The available archaeological and historical data, however, indicate that there was a much larger number of relatively independent communities centered around the numerous smaller image *ahu*. Analyses by Morrison [11] for example, show that the island-wide settlement patterns is characterized by a series of repeated sets of functionally redundant feature classes (e.g., domestic features, earth ovens, walled gardens, etc.) extending over just 300–500 meters in area. This finding is consistent with early ethnohistoric observations. In 1786, for example, the French captain La Pérouse [101] (p.26) notes “the conjectures which may be formed respecting the government of these islanders are, that they compose a single nation, divided into as many districts as there are morais [*ahu*]; because it is to be remarked, that the villages are built near these burying places.” This historical observation reinforces Morrison’s [11] conclusion that Rapa Nui’s settlement pattern is characterized by a series of multiple small communities centered around *ahu* locations.

**Fig 18:**
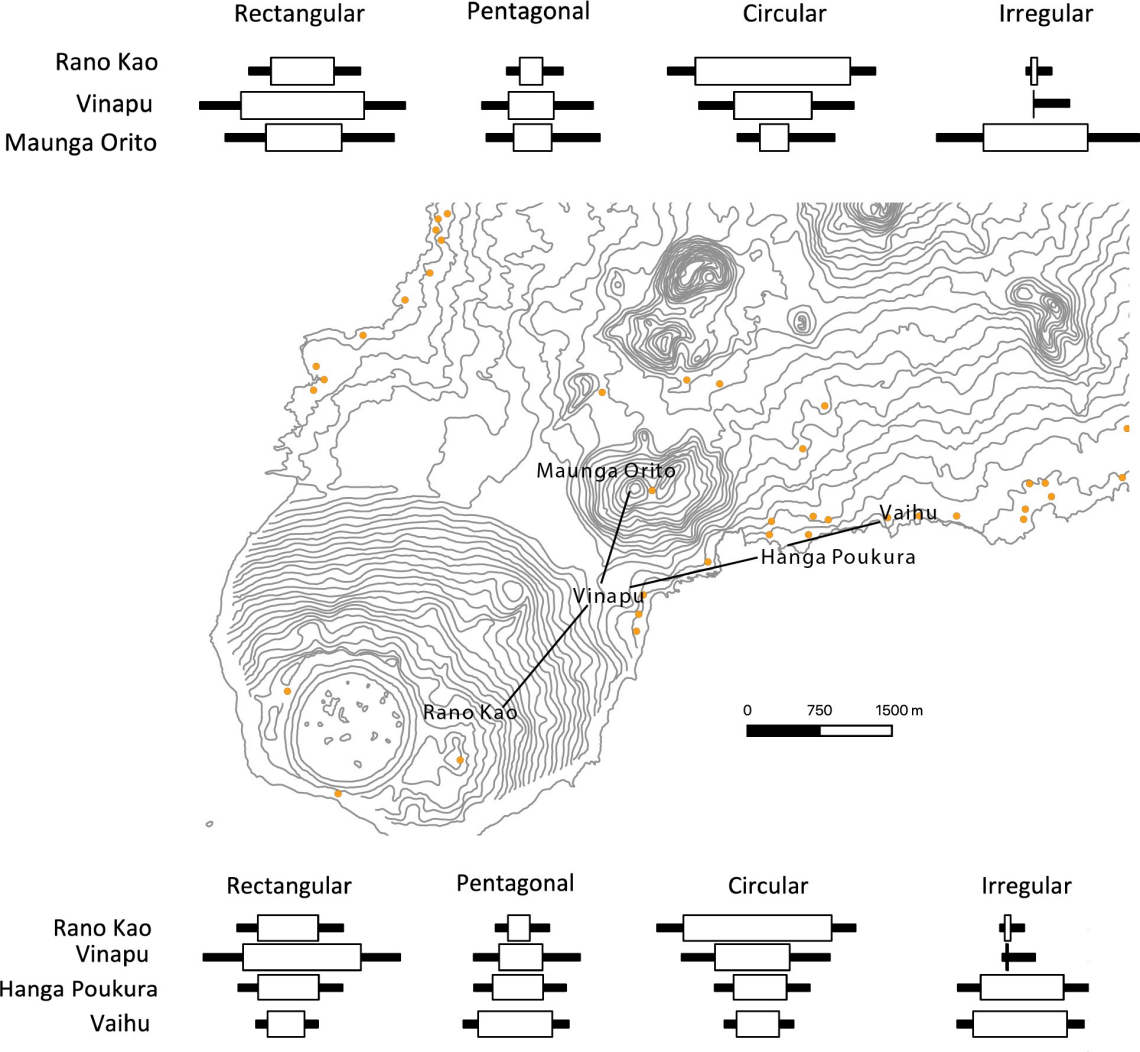
Spatial patterns of seriation solutions of *umu* seriation. Frequency seriation of *umu* shape variability by location. There are two viable solutions to this frequency seriation. Data from Table 1 in McCoy [108].

It is most likely that interaction across the island was nested and varied in spatial distances from extremely local to more island-wide interaction. While communities almost certainly interacted to some degree at larger scales to procure certain raw materials from their source locations, the characteristics of artifacts made from these materials show highly localized scales of cultural transmission. *Pukao*, the large red scoria “hats” associated with at least 50 *moai*, were mostly quarried from a single location at Puna Pau and transported to various locations around the island, and their formal variability is spatially patterned by proximity of their destinations at *ahu* sites [102]. Though more analyses are needed, the *moai* (statues) and *ahu* vary stylistically by spatial proximity over the island, and in the case of *moai* were mostly carved from Rano Raraku statue quarry [e.g., 6,7,100]. Furthermore, several researchers have concluded that the moai quarry shows evidence of multiple independent work areas [e.g., 9,103,104]. Similarly, obsidian was an important raw material available from four source locations on the southwestern part of the island, and obsidian artifacts are abundant in archaeological contexts across the island [105]. While islanders made similar classes of obsidian artifacts, such as stemmed obsidian tools (*mata‘a*), frequency seriations by Lipo et al. [106,107] demonstrate that formal variability in the hafted portion of *mata’a* is strongly spatially biased. Likewise, earth ovens (*umu*) also show patterns of similarity in shape reflecting localized interaction. In the case of *umu* on the southwest sector of Rapa Nui [10,108], and frequency seriation results show spatial patterns indicating traditions of making an *umu* reflect sharing among immediate neighbors (Fig 18). Together, the available data and analyses of formal variability in a range of artifact classes indicate that while islanders did produce similar kinds of artifacts from common raw material source locations, formal variability in material culture shows that information sharing about manufacture styles and techniques was often constricted to small local areas.

Another aspect of Rapa Nui material culture is *rongorongo*, a system of glyphs carved into wooden tablets. The antiquity of *rongorongo* is unknown, although current evidence suggests some of the known tablets likely date to the post-contact period [8,109,110]. The single radiocarbon date from a *rongorongo* tablet collected in 1871 [110] (Beta-184112, 80 +/- 40 BP) calibrates to 1695–1725 cal AD (11.7%) and 1805–1950 cal AD (83.7%) using the SHcal20 calibration curve [111]. Based on ethnographic work by Metraux [8] (pp. 389–409), these glyphs likely represent abstract concepts consistent with mnemonic devices for recording memorized chants of stories or genealogies. As Metraux [8] (p.404) describes, “all the traditions and all the statements of the natives agree that the tablets were associated with memorized chants. … the symbols formed a sort of pictography in the sense that each glyph was associated with a particular sentence or group of words in a chant. The symbols did not correspond exactly to a specific chant, but each tablet could be used with many chants, and several sentences were linked with each image.” Why do we see this form of information transfer on Rapa Nui and not elsewhere in Polynesia [8,109]? While arrangements of knots were used for similar mnemonic purposes elsewhere in Polynesia [109] (p.167-169), these systems are fairly simple compared to the potential informational complexity encoded by the rongorongo scripts and tablets. Our results suggest an intriguing hypothesis that the glyphs of *rongorongo* served as mnemonic devices that were particularly key in encoding, transmitting, and retaining cultural information in Rapa Nui’s precarious environment.

In addition to the evidence for material culture, highly localized interaction is reflected in human remains. Genetic evidence from pre-contact skeletal material points to strong localized interaction. Dudgeon’s [112,113] research using genetic data from human teeth demonstrates a high proportion of similarity at the site and subpopulation level. Dudgeon [112] also shows that patterns of minor and trace elements found in the dental enamel are regionally distinctive, as do other stable isotope analyses [e.g., 114,115]. In his 2008 analysis using Mahalanobis’ posterior classification of human remains, Dudgeon successfully groups instances of human remains by spatial proximity more than eighty percent of the time. This result points to individuals who were largely constrained in their consumption of food and water to localized areas of the island. Analyses of pre-contact skeletal traits show similar localized patterns. For example, variation in non-metric cranial traits reveals strong spatial patterning [116], and the appearance and frequency of rare discrete traits point to limited intra-island gene flow [113]. Gill and colleagues [13,117] note the high frequency of discrete traits in some co-interred individuals on the north and west coasts and for cave internments near *ahu* on the south coast. These discrete features include the bipartite patella and fused Sacroiliac joints. Notably, females show less mobility than males. In a study using minimum genetic distance, Stefan [118] found evidence of greater between-group homogeneity within the male skeletal sample, indicating higher island-wide mobility of males compared to females. Taken together, the biological and archaeological data suggest strongly localized patterns of cultural and genetic transmission. Given the model results, we hypothesize that such locally structured interaction on Rapa Nui would have promoted retention of cultural diversity and richness; an adaptation that mitigated the potentially deleterious effects of drift on this small island with a small overall population.

## Limitations and future directions

Despite its small size, Rapa Nui communities appear to have been relatively small, numerous, and interacted primarily with close neighbors. The adaptive aspects of this highly-localized community patterning may be explained by the cultural transmission model presented: increases in overall network distance were beneficial in the retention of cultural information that might otherwise be at risk from the effects of drift. Cumulative cultural information would have value facing challenges arising beyond the lifespan of single individuals. The adaptive aspects of localized interaction on Rapa Nui may have ensured that cultural diversity and information were not lost among communities that were small and isolated. Such solutions work to optimize group interactions in the creation and retention of innovations. Retaining localized knowledge can be particularly important on small islands where conditions for success may require highly specific knowledge systems [e.g., 119]. In some cases, these knowledge systems may need to be highly localized; an important consideration in planning for uncertainty of climate change for these island locations.

While the archaeological, historical, and biological evidence broadly conforms to the model expectations, there remain limitations in the data that preclude more rigorous model fitting and comparison. While different lines of evidence offer a coherent picture of highly localized genetic and cultural transmission, these broad scale patterns are somewhat at odds with finer scale parameterizations and predictions of the cultural transmission model. The available archaeological data, particularly from abundant lithic artifacts (*mata‘a*) and cooking features (*umu*), are relevant to examine the models’ predictions, but detailed information remains unrecorded or unanalyzed for much of the island. Once larger sample sizes from broader spatial scales become available, promising future research would be to examine the fit between temporal and spatial patterns of artifactual frequency data and the model expectations using Approximate Bayesian Computation or other means of model assessment [e.g., 27,120].

The case we present from Rapa Nui furthers our understanding of the mechanisms that drive changes in cultural diversity more broadly, which remains an important and highly debated issue in cultural evolutionary research. Several pioneering studies proposed models whereby changes in effective population sizes may account for large-scale changes in cultural complexity in human history [e.g., 29,30,121]. These studies, however, have often not explicitly accounted for the fact that humans live in variable social networks, and recent models have shown that population structure is equally important to demography in influencing changes in the diversity and complexity of cultural traits [e.g., 28,50–52]. Here, we provide a model for exploring how changes in the configuration and patterns of interaction among and between subpopulations can dramatically increase diversity as well as retain richness in even relatively small and isolated populations.

## Supporting information

S1 Table Configurations for simulation runs. (DOCX)

S1 File (DOCX)
