# Supplementary material for: Population structure drives cultural diversity in finite populations: A hypothesis for localized community patterns on Rapa Nui (Easter Island, Chile)
Source: PLoS One. 2021 May 12;16(5):e0250690. doi: 10.1371/journal.pone.0250690 (PMC8115772; doi:10.1371/journal.pone.0250690)
Supplement: S1 Table — (DOCX) [file pone.0250690.s001.docx]

**Table S1.** Configurations for Simulation Runs

| **Figure** | **Network Configuration** | **Initial Traits** | **Innovation Rate** | **Simulation Length** | **Population Size** | **Interaction Rate** | **k-values** | **Number of Subpopulations** | **Rewiring Probability** | **Repetitions** |
| --- | --- | --- | --- | --- | --- | --- | --- | --- | --- | --- |
| Figures 3-4 | Watts-Strogatz | 100 | 0.00 | 2000 | 5000 | 0.0001 | 5, 20, 120 | 150 | 0.001 | 10 |
| Figures 5-6 | Watts-Strogatz | 100 | 0.00 | 2000 | 5000 | 0.0001 | 2 | 2, 50, 200 | 0.001 | 10 |
| Figures 7-8 | Watts-Strogatz | 100 | 0.00 | 2000 | 5000 | 0.0001-0.005 | 5-190 | 200 | 0.001 | 5 |
| Figures 9-10 | Watts-Strogatz | 100 | 0.00 | 2000 | 5000 | 0.0001-0.005 | 2 | 5-200 | 0.001 | 5 |
| Figures 11-12 | Watts-Strogatz | 100 | 0.00 | 2000 | 5000 | 0.0001-0.005 | 2 | 5-200 | 0.001 | 5 |
| Figures 13-14 | Rapa Nui Ahu | 100 | 0.00 | 2000 | 5000 | 0.0001 | 2, 50, 140 | 150 | 0.001 | 5 |
| Figures 15-16 | Rapa Nui Ahu | 100 | 0.00 | 2000 | 5000 | 0.00001-0.00005 | 5-125 | 150 | 0.001 | 5 |
