# Supplementary material for: Population structure drives cultural diversity in finite populations: A hypothesis for localized community patterns on Rapa Nui (Easter Island, Chile)
Source: PLoS One. 2021 May 12;16(5):e0250690. doi: 10.1371/journal.pone.0250690 (PMC8115772; doi:10.1371/journal.pone.0250690)
Supplement: S1 File — (DOCX) [file pone.0250690.s002.docx]

Simulations were scripted in Python 3.7. Data from the simulation were used to produce heatmaps using R (4.0.0; R Core Team 2020) and the *ggplot2* package (*v3.3.2*; Wickham 2016). All Python code for the simulation, lists of parameters used in each of the analyses, and R code for producing the paper’s visualizations can be found at <https://github.com/clipo/network-drift>. All of the paper’s analyses and results can be reproduced using the Docker file available at <https://hub.docker.com/repository/docker/mmadsen/network-drift> .
